# The Concerted Action of E2-2 and HEB Is Critical for Early Lymphoid Specification

**DOI:** 10.3389/fimmu.2019.00455

**Published:** 2019-03-18

**Authors:** Thibault Bouderlique, Lucia Peña-Pérez, Shabnam Kharazi, Miriam Hils, Xiaoze Li, Aleksandra Krstic, Ayla De Paepe, Christian Schachtrup, Charlotte Gustafsson, Dan Holmberg, Kristina Schachtrup, Robert Månsson

**Affiliations:** ^1^Department of Laboratory Medicine, Center for Hematology and Regenerative Medicine, Karolinska Institutet, Stockholm, Sweden; ^2^Faculty of Medicine & Faculty of Biology, Center for Chronic Immunodeficiency, Medical Center, University of Freiburg, Freiburg, Germany; ^3^Faculty of Medicine, Institute of Anatomy and Cell Biology, University of Freiburg, Freiburg, Germany; ^4^Lund University Diabetes Center, Lund University, Malmö, Sweden; ^5^Hematology Center, Karolinska University Hospital, Stockholm, Sweden

**Keywords:** E-protein, lymphoid specification, hematopoiesis, humoral immunity, evolution

## Abstract

The apparition of adaptive immunity in *Gnathostomata* correlates with the expansion of the E-protein family to encompass E2-2, HEB, and E2A. Within the family, E2-2 and HEB are more closely evolutionarily related but their concerted action in hematopoiesis remains to be explored. Here we show that the combined disruption of E2-2 and HEB results in failure to express the early lymphoid program in Common lymphoid precursors (CLPs) and a near complete block in B-cell development. In the thymus, Early T-cell progenitors (ETPs) were reduced and T-cell development perturbed, resulting in reduced CD4 T- and increased γδ T-cell numbers. In contrast, hematopoietic stem cells (HSCs), erythro-myeloid progenitors, and innate immune cells were unaffected showing that E2-2 and HEB are dispensable for the ancestral hematopoietic lineages. Taken together, this E-protein dependence suggests that the appearance of the full *Gnathostomata* E-protein repertoire was critical to reinforce the gene regulatory circuits that drove the emergence and expansion of the lineages constituting humoral immunity.

Large *Eumetazoan* rely on an efficient system of innate and adaptive immune cells to survive and reach reproductive age (1–3). The different cells of the hematopoietic system are all generated from hematopoietic stem cells (HSCs) (4). Lymphoid specification is initiated in lymphoid primed multipotent progenitors (LMPPs) that start to express genes associated with adaptive immune cells (5, 6). LMPPs subsequently give rise to common lymphoid precursors (CLP) (7). Within the heterogeneous CLP population, the LY6D^+^ fraction is further specified toward a B-lineage fate (8, 9) and contains the first B-lineage committed cells that subsequently give rise to mature B-cells (9, 10). Early lymphoid precursors leave the bone marrow to seed the thymus where they further develop into early T-cell progenitors (ETP) that give rise to mature T-cells (11). Similarly, the innate immune cells develop from different progenitors within the myeloid branch (12, 13), while natural killer (NK) cells and part of the dendritic cells (DC) develop from the CLP (7, 14).

The origin of the *Gnathostomata* (jawed vertebrate) hematopoietic system can be traced far back in evolutionary history with phagocytic and cytotoxic innate immune cells being found across the *Bilateria* (15) and the erythroid/megakaryocyte lineages appearing in the *Agnatha* (16). Similarly, lymphoid-like cells are present in the *Agnatha* (17), *Urochordata* (18), and *Cephalochordata* (19). However, while genes intimately associated with adaptive immunity—including RAG (20, 21), histocompatibility genes (22, 23), and immune type receptors (22, 24, 25)—are found in lower *Deuterostomata*, B- and T-cells mediated adaptive immunity emerged only in the *Gnathostomata*. The appearance of new transcription factor (TF) genes drive the apparition of novel cell types (26). The appearance of adaptive immunity in the *Gnathostomata* correlates with a dramatic increase in TF genes (1, 27). As part of this expansion, the full *Gnathostomata* basic helix-loop-helix E-protein family (28, 29) consisting of E2A (Tcf3), HEB (Tcf12), and E2-2 (Tcf4) emerged.

It has been proposed, that E2A is more closely related to the ancestral E-proteins while E2-2 and HEB are less evolutionarily conserved and display expression patterns more restricted to vertebrate-specific structures (29, 30). This suggests that E2A should govern ancestral functions while HEB and E2-2 should govern novel functions that emerged concomitantly to the rise of the *Gnathostomata*. In line with this, E2A is the only E-protein reported to impact HSC function and the development of the myeloid- and erythro/megakaryocytic lineages (31–35). In contrast, all the E-proteins promote development of B- and T-cells (32, 36–47). The potential role of E2-2 in stem- and progenitor cells remains largely unexplored.

Here we confirm that E2-2 and HEB are evolutionary related and we found that their coordinated action is critical for the development of early lymphoid progenitors. Mice lacking both E2-2 and HEB display an almost complete block in B-cell development at the level of the CLP and the few generated immature B-cells preferentially develop into marginal zone (MZ) B-cells. Similarly, we found T-cell development to be perturbed, resulting in reduced numbers of CD4 T-cells and increased numbers of γδ T-cells. In contrast, HSCs, erythro-myeloid development and the generation of innate immune cells were unperturbed. Together, this suggests that E2-2 and HEB are dispensable for ancestral hematopoietic lineages and that the appearance of the full *Gnathostomata* E-protein repertoire promoted the apparition of humoral immunity.

## Materials and Methods

### Animal Studies

To generate mice lacking specific E-proteins in the hematopoietic system, Vav-iCre (48) was used in combination with conditional (floxed) E2-2 (49), HEB (44), and E2A (50) alleles. Mice were maintained on a C57BL/6 background and analyzed at 8–14 weeks of age. Animal studies were approved by the local ethics committee (ethical approval number S16-15).

### Preparation of Cells and Flow Cytometry

Bones, spleen, and thymus were dissected, crushed in PBS with 2% FCS and cells were collected after passing through a 70 μm filter. They were then Fc-blocked (CD16/32; 93) and stained with combinations of the antibodies Sca1 (D7), CD105 (MJ7/18), CD41 (MWReg30), CD48 (HM48-1), CD3 (145-2C11), CD4 (RM4-5), CD8 (53-6.7), B220 (RA3-6B2), NK1.1 (PK136), Mac1 (M1/70), Gr1 (RB6-8C5), TER119 (TER-119), CD150 (TCF15-12F12.2), CD117 (2B8, eBioscience), CD127 (A7R34), CD44 (IM7), CD25 (PC61.5, eBioscience), CD19 (1D3, eBioscience), TcRβ (H57-597, eBioscience), TcRγδ (GL3, eBioscience), Ly6C (AL-21), Ly6G (1A8), MHCII (M5/114.15.2), CD11c (N418), PDCA1 (927), Ly6D (49H4), Flt3 (A2F10), IgD (11-26c.2a), and IgM (11/41, eBioscience). All antibodies were purchased from BD Biosciences unless otherwise indicated. Propidium iodide (PI) was utilized to discriminate dead cells. For hematopoietic stem and progenitor cell isolation, cells were subjected to lineage depletion using Dynabeads sheep anti rat IgG (Life Technologies) together with TER119, CD19, CD3, Gr1, and CD11b antibodies prior to staining. Analysis and cell sorting was performed primarily on an LSR Fortessa and FACSAria IIu (BD Biosciences). Analysis of data was done using the Flowjo 9.9.6 software (Flowjo).

### Phylogenetic Analysis

The cDNA and amino acids sequences of the E-proteins from analyzed organisms were obtained through the E-ensembl repository (51). See Table S1 for the sequences used in this study. Phylogenetic trees were constructed with MEGA7 (52) selecting the Maximum Likelihood method based on the Tamura-Nei model; creating initial tree(s) using the Neighbor-Joining and BioNJ algorithms; and using a Gamma distribution with invariant sites. All positions in the cDNA and amino acids sequences (including gaps) were considered when constructing the trees. To assess the support of each node, the tree was bootstrapped 500 times.

### RNA Sequencing and Analysis

5-10 × 10^3^ cells were FACS-sorted into buffer RLT with β-mercaptoethanol and total RNA extracted using RNeasy Micro Kit (Qiagen, Hilden, Germany) with on-column DNase I treatment. Strand specific RNAseq libraries were prepared using the TotalScript™ RNA-seq kit (Epicenter, Madison, WI) together with custom made Tn5 (transposase). Barcoded libraries were pooled and pair-end sequenced (2 × 50 cycles) using the Illumina platform (NextSeq500, Illumina, San Diego, CA).

RNAseq reads were mapped using STAR (53), reads in exons quantified using HOMER (54) and significant changes identified using EdgeR. Principal component analysis (PCA) analysis and display was performed using R (v3.3.3). For details see Supplemental Materials and Methods.

### Pro B Cell Expansion Cultures and Fixation

B220^+^ cells were isolated from bone marrow of ER-Cre mice (55) using magnetic cell separation (Miltenyi Biotec), expanded for 6 days in the presence of IL-7 and SCF to obtain pro B-cells. Pro B-cells were subsequently retrovirally transduced with Bcl-2, expanded for seven additional days with 5 μM 4-Hydroxytamoxifen in the medium during the last 72 h. Pro B-cells were fixed using EGS (1.5 mM for 30 min) in combination with PFA (1% for 10 min) and stored as pellets in −80°C.

### ChIP Sequencing and Analysis

In brief, fixed pro B-cells were thawed, resuspended in SDS lysis buffer, sonicated, and lysates subjected to ChIP by adding dynabeads pre-loaded with either antibodies against E2A, HEB, or E2-2. ChIPed chromatin was washed and libraries prepared using reverse crosslinking in conjunction with adapter ligation using the ThruPLEX DNA-seq kit (Rubicon Genomics) or using the high-throughput ChIPmentation approach (tagmentation followed by direct amplification of libraries without prior DNA purification) (56). For details see Supplemental Materials and Methods. Libraries were sequenced using the HiSeq2000 or NextSeq500 platforms (Illumina).

ChIPseq reads were mapped using bowtie2 (57). Identification of peaks, peak overlaps and motif enrichment/identification analysis was done using HOMER's findPeaks, mergePeaks, and findMotifsGenome.pl, respectively (54). Visualization was done using the UCSC genome browser. For details see Supplemental Materials and Methods.

## Results

### E2-2 and HEB Are Evolutionarily Related

To investigate the evolutionary history of the E-proteins in *Bilateria* in light of recent sequencing data, we analyzed similarities between cDNA and amino acid sequences of the E-proteins across *Animalia* (Figures S1A–D). The E-proteins of *Protostomata* and non-gnathostome *Deuterostomata* clustered together (Figure S1B) and displayed relatively high sequence divergence (Figure S1C) on the cDNA level. The *Gnathostomata* E-proteins, in contrast, formed a separate clade (Figure S1B) and displayed comparably higher similarity (Figure S1C). Within the *Gnathostomata* clade, E2-2, and HEB formed a separate branch from that of E2A (Figure S1B). Similar results were obtained from the analysis of the amino acid sequences (Figure S1D). This confirms the closer evolutionary relation between E2-2 and HEB in *Gnathostomata* (29, 58). It could hence be hypothesized that E2-2 and HEB are functionally related and together support the development of cell lineages specific to the jawed vertebrate hematopoietic system.

### Deletion of E2-2 and HEB Does Not Impact HSCs and Erythro-Myeloid Progenitors

**Figure 1:**
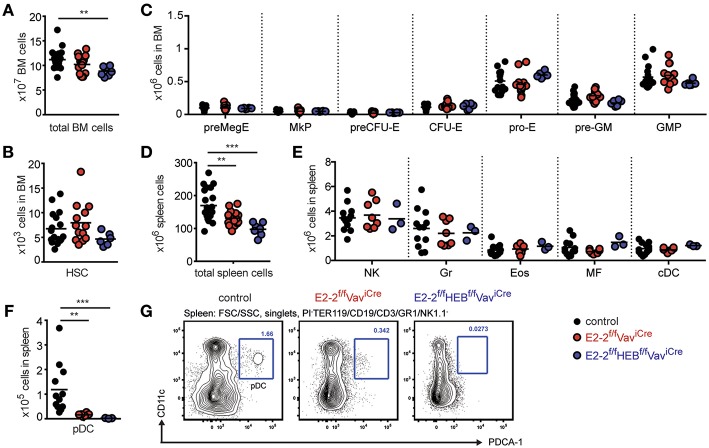
E2-2 and HEB are dispensable for ancestral lineages. Absolute number of total cells **(A)**, hematopoietic stem cells (HSCs) **(B)**, and erythro-myeloid progenitors **(C)** in bone marrow. Absolute number of total cells **(D)**, innate immune cells **(E)** (Gr, granulocytes; Eos, eosinophiles; MF, macrophages; cDC, conventional dendritic cells) and plasmacytoid dendritic cells (pDC) **(F)** in spleen. For gating strategies for cell types in panel E-F and H see Figures S3A–C. **(G)** Gating strategy for identification of pDCs. Prior gating is indicated above the FACS plots. Color symbols utilized throughout the figure to indicate the genotype of analyzed mice are shown in the bottom right corner. Significance was calculated using the Mann-Whitney U test with ^**^ and ^***^ indicating *p*-values < 0.01 and < 0.001, respectively.

To investigate the role of E2-2 and HEB in hematopoiesis, we used the Vav^iCre^ mouse strain to mediate conditional deletion of E2-2 (E2-2^f/f^Vav^iCre^) or E2-2 together with HEB (E2-2^f/f^HEB^f/f^Vav^iCre^) in the hematopoietic system. Cre mediated deletion of the floxed exons was verified in RNAseq data from FACS sorted cells (Figures S2A–C). We observed a difference in bone marrow (BM) cellularity only between littermate control mice (lacking Vav^iCre^) and E2-2^f/f^HEB^f/f^Vav^iCre^mice (Figure 1A). However, no differences were observed in the number of HSCs (Figure 1B, Figure S3A), megakaryocyte, erythroid, and myeloid progenitors (Figure 1C, Figure S3B). While being expressed in the HSC and erythro-myeloid progenitors (Figure S2D), E2-2 and HEB are hence dispensable for the maintenance and generation of these cell types in steady state (32). This leaves E2A as the sole E-protein needed for HSCs and erythro-myeloid development (31).

### Generation of Innate Immune Cells Except pDCs Are Unaffected by E2-2 and HEB Deletion

While the total number of spleen cells was reduced in E2-2^f/f^HEB^f/f^Vav^iCre^mice (Figure 1D), no effect was observed on the number of mature myeloid cells (Figure 1E, Figure S3C). This further support that E2-2 and HEB are dispensable for myelopoiesis. Similarly, natural killer (NK) cell numbers were unaffected (Figure 1E, Figure S3C). In contrast, plasmacytoid dendritic cells (pDC) were reduced by 86% by the deletion of E2-2 and by 98% with the additional removal of HEB (Figures 1F,G) indicating a previously unrecognized dependence on HEB for pDC development (59).

### E2-2 and HEB Are Needed for Proper Generation of B-Cells and CD4 T-Cells

**Figure 2:**
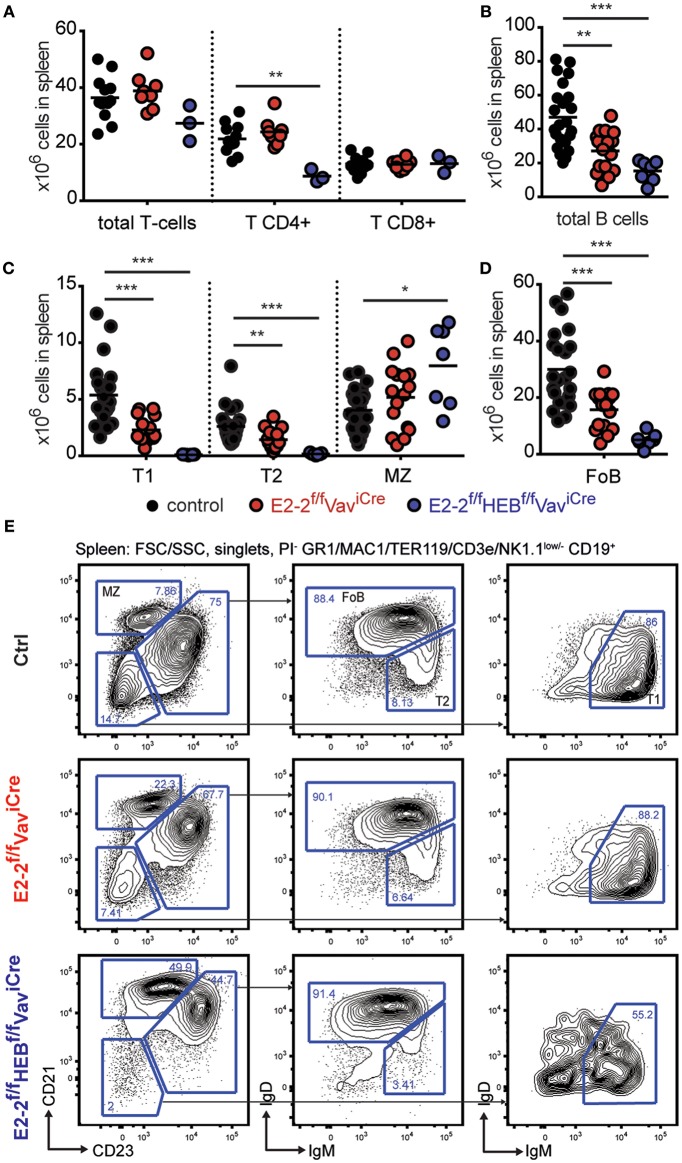
E2-2 and HEB are needed for proper B-cell and CD4 T-cell generation. **(A)** Absolute number of T-cells, CD4 T-cells, and CD8 T-cells in spleen. For T-cell gating strategy see Figure S3C. Absolute number of B-cells **(B)** and B-cell subsets **(C,D)** (including: T1, transitional 1-, transitional 2-; MZ, marginal zone; and FoB, follicular B-cells) in spleen. **(E)** Gating strategy for identification of B-cell subsets. Prior gating is indicated above the FACS plots. Color symbols utilized throughout the figure to indicate the genotype of analyzed mice are shown below panels **(C,D)**. Significance was calculated using the Mann-Whitney U test with ^*^, ^**^, and ^***^ indicating *p*-values < 0.05, < 0.01, and < 0.001, respectively.

As the total number of spleen cells was reduced (Figure 1D), we next investigated the effect of deletion of E2-2 and HEB on the adaptive immune cells. The number of T-cells in the spleen was not impacted by the depletion of E2-2, but E2-2^f/f^HEB^f/f^Vav-iCre mice displayed a 60% reduction in CD4 T-cells (Figure 2A). The total number of B-cells in spleen was reduced by 42% by the deletion of E2-2 and by 68% when HEB was additionally depleted (Figure 2B). This reduction reflected a sharp drop in transitional- and follicular B-cells (FoB) while marginal zone (MZ) B-cell numbers remained unaffected in E2-2^f/f^Vav-iCre and increased in E2-2^f/f^HEB^f/f^Vav-iCre mice (Figures 2C–E). This suggests that the removal of E2-2 and HEB heavily promoted the generation of MZ from transitional B-cells at the expense of FoB (60, 61). Taken together, these data show that cell lineages with ancestral functions (erythro-myeloid and cytotoxic cells) (15, 16, 62–65) are independent of E2-2 and HEB. In contrast, cell lineages central to adaptive (humoral) immunity (B- and CD4 T-cells) or at the interphase between innate and adaptive immunity (pDC) are dependent on E2-2 and/or HEB.

### The Development of CD4 and γδ T-Cells Is Perturbed by the Loss of E2-2 and HEB

**Figure 3:**
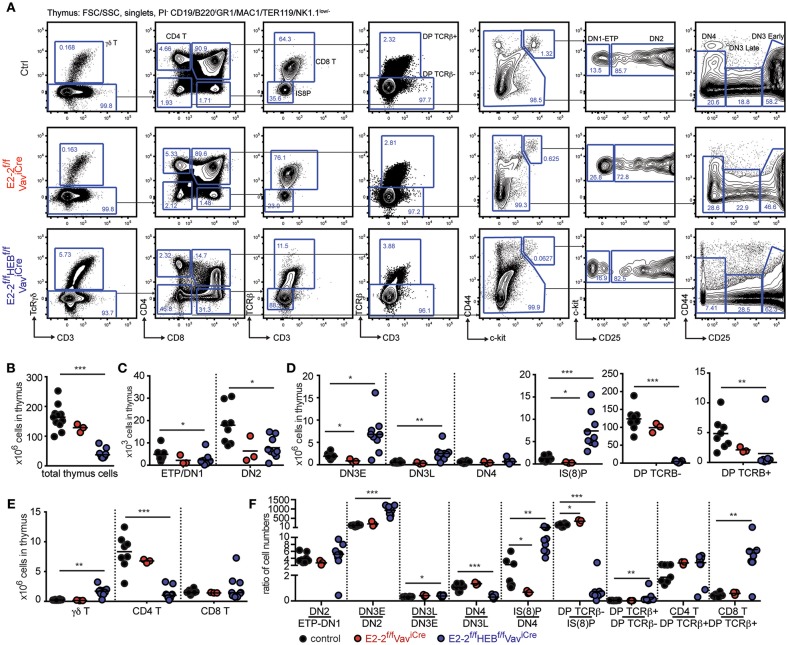
Maturation of CD4 and γδ T-cells is perturbed by the loss of E2-2 and HEB. **(A)** Gating strategy for identification of T-cell developmental stages. Absolute numbers of: total cells **(B)**; ETP/DN1 and DN2 **(C)**; DN3E, DN3L, DN4, IS(8)P, DP TCRβ^−^, and DP TCRβ^+^ **(D)**; and γδ T-, CD4 T- and CD8 T-cells **(E)** in thymus. Prior gating is indicated above the FACS plots. **(F)** Cell number ratios for each consecutive stage of development as a fraction of the prior stage. Each dot represents the ratio for one individual animal. Color symbols utilized throughout the figure to indicate the genotype of analyzed mice are shown at the bottom of the figure. Significance was calculated using the Mann-Whitney U test with ^*^, ^**^, and ^***^ indicating *p*-values < 0.05, < 0.01, and < 0.001 respectively.

With E2-2^f/f^HEB^f/f^Vav^iCre^ mice displaying decreased CD4 T-cells in the periphery (Figure 2A), we next investigated T-cell development in the thymus (Figure 3A). Depletion of E2-2 had no effect on thymic cellularity (Figure 3B). However, E2-2^f/f^Vav^iCre^ mice displayed a visible decrease in ETPs and DN2 followed by decreases in DN3E and IS(8)P (Figure 3D). In line with previous studies of HEB knock-out animals (36), the additional deletion of HEB perturbed T-cell development (Figure 3A) with decreased total cellularity in thymus (Figure 3B); significant decreases in ETP and DN2 (Figure 3C); increases in DN3E, DN3L, and IS(8)P (Figure 3D); but markedly reduced DPs (Figure 3D). In spite of the marked reduction in DPs, CD8 T-cells were present in normal numbers (Figure 3E) while CD4 T-cells were severely reduced (>85%). Additionally, E2-2^f/f^HEB^f/f^Vav^iCre^ animals displayed markedly increased (6-fold) γδ T-cells numbers (Figure 3E) (36, 66).

Looking closer at the progressive generation of cells from the prior stage in each developmental transition in E2-2^f/f^HEB^f/f^Vav^iCre^ mice, the altered cell numbers observed were mirrored by increased generation of DN3E, DN3L, and IS(8)P before a sharp drop in the generation of DP TCRβ^−^cells (Figure 3F). While few in numbers, DP TCRβ^+^ were generated in increased numbers with CD4 and CD8 T-cells subsequently being generated at a normal and increased ratio, respectively (Figure 3F). Taken together these results demonstrate that E2-2 and HEB are needed for normal thymopoiesis.

### E2-2 and HEB Are Critical for Lymphoid Specification and B-Cell Development

**Figure 4:**
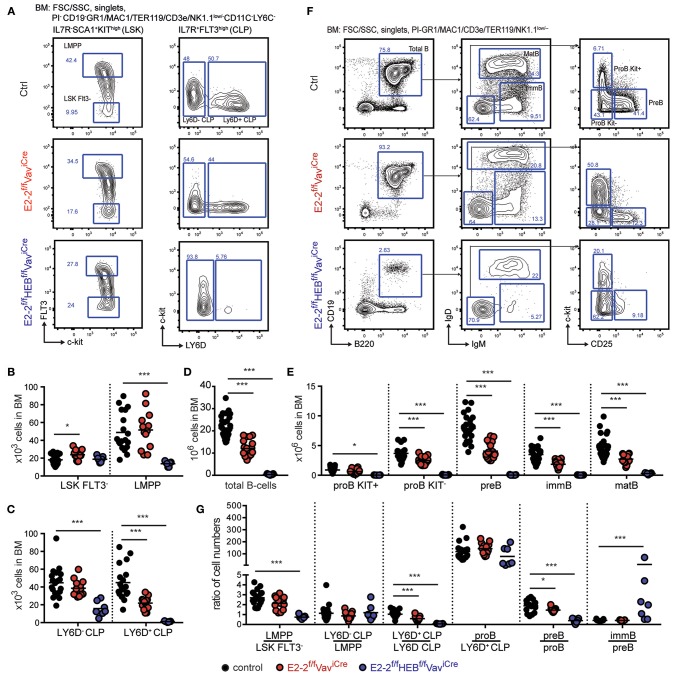
The combined activity of E2-2 and HEB are critical for the generation of lymphoid progenitors and B-lineage cells. **(A)** Gating strategy for identification of stem- and lymphoid progenitors. Prior gating is indicated above the FACS plots. **(B,C)** Absolute numbers of stem- and lymphoid progenitors indicated cell types in BM. Absolute numbers of total B-cells **(D)** and each B-cell developmental stage **(E)** in BM. **(F)** Gating strategy for identification of total B-cells and B-cell developmental stages. Prior gating is indicated above the FACS plots. **(G)** Cell number ratios for each consecutive stage of development as a fraction of the prior stage. Each dot represents the ratio in an individual animal. Color symbols utilized throughout the figure to indicate the genotype of analyzed mice are shown below panel G. Significance was calculated using the Mann-Whitney U test with ^*^ and ^***^ indicating *p*-values < 0.05 and < 0.001 respectively.

The development of LMPPs and CLPs constitute the first steps of lymphoid specification (5, 7, 9). Depletion of E2-2 alone did not significantly affect the number of LMPPs and Ly6D^−^CLPs (Figures 4A–C). However, the number of B-cell specified LY6D^+^CLPs (8, 9) was reduced by half (Figure 4C). Correspondingly, total B-cells were reduced by 43% (Figure 4D) with each stage in B-cell development displaying a 30–50% reduction (Figures 4E,F). The additional loss of HEB, in contrast, lead to a 70% decrease in the number of LMPPs and LY6D^−^CLP (Figures 4A–C). In addition, the E2-2^f/f^HEB^f/f^Vav^iCre^ mice strikingly displayed a near complete loss of LY6D^+^CLP (>98% reduction) (Figures 4A,B). This phenotype is similar to what has previously been reported for E2A knock-out (32). Accordingly, B-cell numbers were severely reduced (Figure 4D) with a >99% reduction in cell-numbers of maturing B-cells (Figures 4E,F) and a 94% reduction in mature B-cells (Figures 4E,F) in BM.

To analyze more closely the impact of E2-2 and HEB depletion on developmental transitions, we plotted cell number ratios at each consecutive stage of development as a fraction of the prior stage (Figure 4G). E2-2 deficient mice only displayed a modest decrease in the generation of LY6D^+^CLP from LY6D^−^CLP while the consecutive generation of CD19^+^ developmental stages remained unaffected (Figure 4G). This indicates that E2-2, similarly to what has been reported for HEB deficient animals (32), is important for the LY6D^−^ to LY6D^+^ CLP transition but largely dispensable for BM B-cell maturation. This is in line with the expression data showing that early lymphoid progenitors (LMPPs and CLPs) expressed similar levels of E2-2, HEB and E2A while HEB and E2A were the predominantly expressed E-proteins in B-lineage cells (Figure S2D). The combined deletion of E2-2 and HEB severely impacted the generation of LMPPs, LY6D^+^CLP and pre-B while, at the same time, seemingly increasing the generation of immature B-cells (Figure 4G). Interestingly, the generation of proB cells from the few remaining LY6D^+^CLP was unaffected (Figure 4G), indicating that B-lineage commitment at this stage is unperturbed by the lack of E2-2 and HEB. Together, this demonstrates that the collaboration of E2-2 and HEB is critical for the generation of early lymphoid progenitors and the development of B-cells in the BM.

### Expression of the Early Lymphoid Program Is Disrupted by Removal of E2-2 and HEB

**Figure 5:**
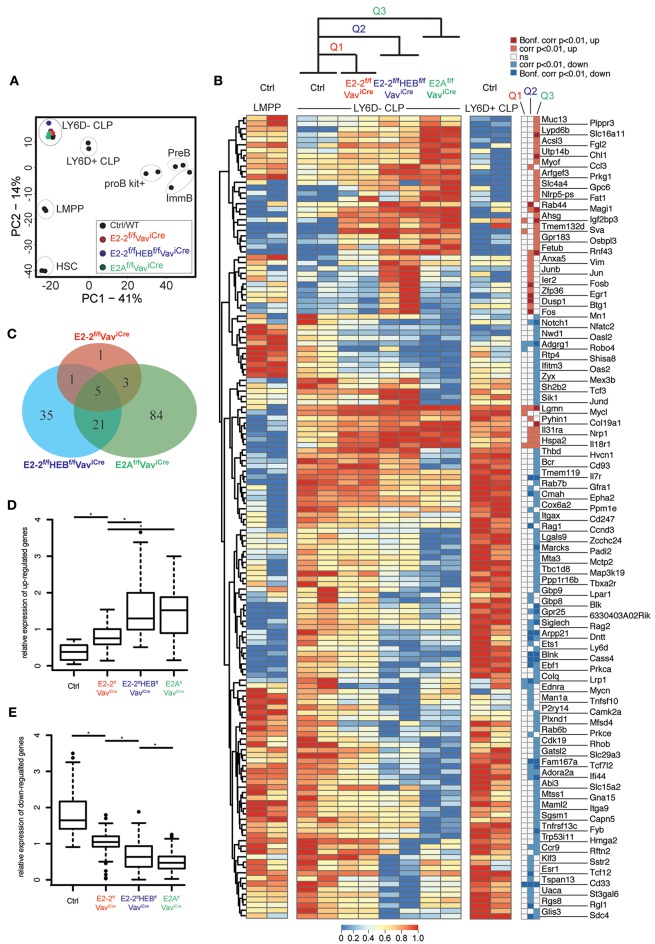
Loss of E-protein activity disrupts the early lymphoid transcriptional program. **(A)** Principal component analysis of RNAseq data. **(B)** Hierarchically clustered heatmap showing the (row normalized) expression of genes with significant (adjusted *p*-value < 0.01 calculated by EdgeR) changes in LY6D^−^CLPs from E2-2^f/f^Vav^Cre^ (Q1), E2-2^f/f^HEB^f/f^Vav^Cre^ (Q2) or E2A^f/f^Vav^Cre^ (Q3) as compared to control (ctrl). Significance of expression changes in each comparison (Q1-3) is indicated to the right of the heatmap. **(C)** Venn diagrams showing the overlap between the significant expression changes in the indicated genotypes. Relative expression change (mean expression is set to one for each gene) of up-regulated **(D)** and down-regulated genes **(E)** from Q1-Q3. ^*^indicates a *p*-value < 0.002 calculated using the Mann-Whitney U test.

To better understand the mechanisms behind the impaired generation of the B-cell specified LY6D^+^CLPs and to understand how it relates to the similar phenotype observed in mice lacking E2A (32), we characterized the transcriptional profiles of LY6D^−^CLPs remaining in E2-2^f/f^Vav^iCre^, E2-2^f/f^HEB^f/f^Vav^iCre^, and E2A^f/f^Vav^iCre^ mice using RNAseq (see Table S2 for sample information). Principal component analysis (PCA) of full expression profiles showed that LY6D^−^CLP cells from the different genotypes form a distinct cluster and hence represent the same population of cells regardless of disrupted E-protein gene(s) (Figure 5A). A total of 150 genes displayed significant (corrected *p*-value < 0.01) expression changes in LY6D^−^CLPs in one or more of the analyzed strains (Figure 5B). As expected, B-lineage related genes (including Ebf1, Blnk, Blk, Ets1, Dntt, Notch1, Rag1, and Rag2) were severely affected (Figure 5B) and overall the gene set was functionally associated with lymphocyte differentiation and signaling (Figure S4). While the majority of genes did not show highly significant changes in all genotypes (Figures 5C,D), the overall pattern of the expression changes was highly similar in E2-2^ff^HEB^ff^Vav^iCre^ and E2A^ff^Vav^iCre^ mice (Figures 5B,D,E). The modest expression changes observed in the E2-2^ff^Vav^iCre^ mice were often concordant to those observed in E2A^ff^Vav^iCre^ mice and E2-2^ff^HEB^ff^Vav^iCre^ mice (Figures 5B,D,E). Taken together, this shows that E2-2, HEB and E2A largely reinforce the same gene network in early lymphoid progenitors while having variable impact on the expression of individual genes.

### E-proteins Display Partly Overlapping Association With Chromatin

**Figure 6:**
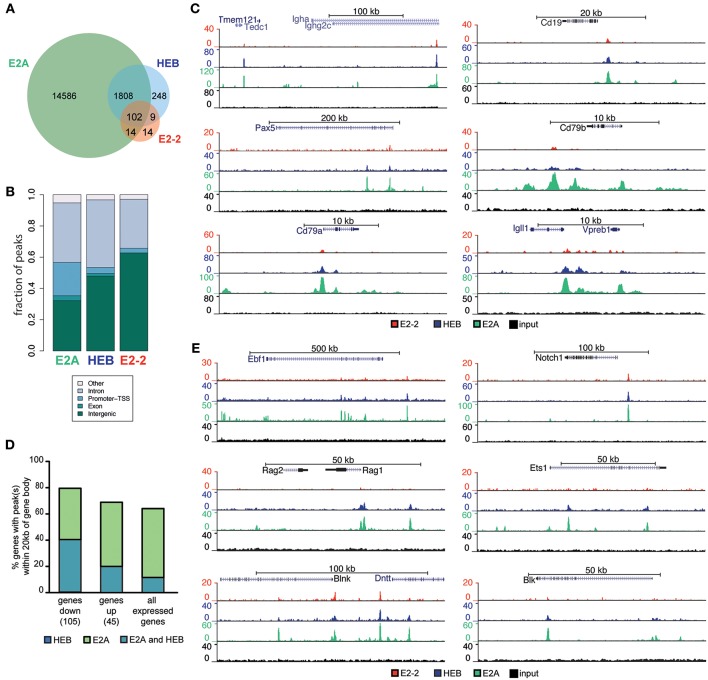
E2-2, HEB and E2A have partly overlapping binding patterns. **(A)** Number and overlap of identified E2-2, HEB, and E2A peaks in proB cells. **(B)** Genomic localization of identified peaks. **(C)** Genome browser tracks showing E-protein binding near central B-lineage genes. **(D)** Percentage of genes in the E-protein dependent early lymphoid program (up/down regulated in Figure 5B) and all expressed genes in LY6D^−^CLPs that have HEB and E2A peaks within the gene body ±20 kb. **(E)** Genome browser tracks showing E-protein binding near genes that were regulated by E-proteins in LY6D^−^CLPs.

To further understand the mechanisms through which E2-2 and HEB control B lymphopoiesis, we analyzed the binding pattern of E2A, HEB and E2-2 in pro B-cells using ChIP-seq in conjunction with adapter ligation (E2A) or high-throughput ChIPmentation (56) (HEB and E2-2) (see Table S2 for sample information). Library preparation using adapter ligation and tagmentation (ChIPmentation) has previously been shown to produce highly comparable results (67). We identified 16510 E2A, 2167 HEB and 139 E2-2 high quality peaks (peak score ≥10) (Figure 6A, Figure S5A). E2-2 is the lowest expressed E-protein in pro B-cells (Figure S2D) and the relatively low enrichment of E2-2 (most peaks have a peak score < 10, Figure S5A) limits accurate peak calling. Hence, E2-2 binding is likely underestimated. Peaks from the three E-protein ChIP experiments displayed highly significant enrichment of E-protein DNA binding motifs (Figure S5B).

E2A mostly bound regions without identified HEB and E2-2 peaks (Figure 6A, Figure S5C) while the majority of E2-2 and HEB peaks overlapped with E2A peaks (Figure 6A, Figure S5C). Interestingly while rare, sites with clearly biased E-protein binding could be identified (Figure S5C). Most binding sites identified where localized in intronic and intergenic regions (Figure 6B). In contrast to the other E-proteins, a significant fraction of E2A peaks were also found in promoter regions (Figure 6B). No difference was observed in the E-protein DNA binding motifs of HEB and/or E2A, with the most enriched motifs containing the same bHLH motif core (CAGCTG) (68). The most enriched motif in E2A peaks was present amongst the motifs found in the peaks common to E2A and HEB (Figure S5D). Genes central to B-lineage, including IgH, CD19, Pax5, CD79a, CD79b, Igll1, and VpreB, were bound by the three E-proteins (Figure 6C). A similar pattern was found for genes that are part of the E-protein-dependent early lymphoid program in LY6D^−^ CLPs (Figure 5B), including Ebf1, Rag1, Rag2, Blnk, Dntt, Notch1, and Blk (Figures 6D,E, Figure S5E). These regions at the center of the lymphoid and B-lineage programs, bound by E2A and HEB, presented a higher enrichment for Ebf and Pax DNA binding motifs compared to regions with only detectable E2A peaks (Figure S5F).

Taken together this further supports the notion that E2-2 and HEB critically reinforce the action of E2A on gene regulatory circuits critical to early lymphoid specification and B-cell development.

## Discussion

Phylogenetic analysis of the E-proteins suggests that the *Gnathostomata* E-protein family arose through two subsequent duplications (29, 58). Interestingly, analysis of the structure of the E-protein loci in non-*Gnathostomata* argues for the original *Gnathostomata* E-protein being structurally similar to the present HEB/E2-2 loci (58). The initial duplication generated two E-protein loci that diverged into the proto-E2-2/HEB and E2A loci. The second duplication subsequently gave rise to the E2-2 and HEB loci as well as two E2A loci out of which one was eventually lost (58, 69). Being evolutionarily related, E2-2 and HEB could hence support similar functions. The E-proteins play critical roles in all the hematopoietic lineages (31, 32, 36, 38, 40–46, 59–61, 70, 71). However, the function of E2-2 in early hematopoietic development and its concerted action with the evolutionarily related E-protein HEB has not been thoroughly addressed.

The B-cell developmental pathway has been shown to critically rely on E2A and HEB, both TFs needed for the generation of LY6D^+^CLPs (32). We similarly found that the loss of E2-2 impaired the generation of LY6D^+^CLPs while earlier progenitors (including LMPPs and LY6D^−^CLPs) were unaffected. Further development of B-cells from LY6D^+^CLPs was unperturbed with the subsequent stages appearing at expected ratios. This indicates that the reduced number of B-cells is primarily caused by the impaired LY6D^−^ to LY6D^+^ transition within the CLP compartment.

Interestingly, the combined deletion of E2-2 and HEB had a direct additive effect resulting in a near complete developmental block at the LY6D^−^CLP stage and dramatically reduced B-cell numbers. Hence, E2-2 and HEB together have a critical and previously unrecognized role in supporting early lymphoid development in the BM. This phenotype is reminiscent of the one observed in E2A knockout animals (32). In line with this, the genes of the early lymphoid program were associated with combined E2A and HEB binding. This further supports the notion that all three E-proteins, to a large extent, reinforce the same gene regulatory circuit in CLPs (32).

The dramatic reduction of BM B-lymphopoiesis in E2-2^ff^Vav^iCre^ and E2-2^ff^HEB^ff^Vav^iCre^ animals was mirrored in reduced numbers of transitional (T1 and T2) B-cells and reduced FoBs. In contrast MZ B-cell numbers were maintained in E2-2^ff^Vav^iCre^ mice or even expanded in E2-2^ff^HEB^ff^Vav^iCre^mice. This, arguing that MZ B-cells are generated at the expense of FoBs, confirming that the levels of all three E-proteins are critical to maintain a normal MZ to FoB ratio [60, 61].

E2-2^ff^Vav^iCre^ and E2-2^ff^HEB^ff^Vav^iCre^ mice displayed, on average, a 60% reduction in ETPs. Hence, while at a reduced level, E2A alone is sufficient to maintain thymic seeding in the absence of the other E-proteins (38, 45). T-cell development downstream of the ETP was significantly perturbed only in E2-2^ff^HEB^ff^Vav^iCre^ animals resulting in reduced CD4 T-cells and increased generation of γδ T-cells. This phenotype is similar to the one observed in the HEB KO mice (36). Hence, this suggests that E2-2 has limited impact on adult thymopoiesis (59).

In line with previous findings, the number of pDCs was greatly reduced in mice lacking E2-2 (59, 72–74). However, we only observed a block in pDCs generation after the additional deletion of HEB (E2-2^ff^HEB^ff^Vav^iCre^). Hence, while E2-2 is clearly the main E-protein needed for pDC development, this indicates that HEB to a limited extent can support pDC development and cooperate with E2-2 in the pDC lineage.

In contrast to the clear impact of E2-2 and HEB on cells involved in humoral immunity (B- and CD4 T-cells), the loss of these TFs did not affect HSC numbers, erythro-myeloid progenitors nor the production of the major innate immune cells lineages including granulocytes, macrophages, and natural killer cells. Similarly the cytotoxic branch of adaptive immunity (CD8 T-cells) was unaffected.

Taken together, this suggests that E2-2 and HEB—in contrast to E2A (31, 33–35)—are dispensable for lineages with ancestral functions. Functionally, this suggests that E2A, while structurally being the most divergent E-protein locus (58), maintains ancestral hematopoietic functions in addition to having been co-opted for lymphoid development and B-cell development in particular.

Until recently, it was hypothesized that the adaptive immune system arose from an evolutionary “big bang” at the speciation of the *Gnathostomata* (1). However, advances in genome sequencing of lower *Deuterostomata* has shifted this dogma by describing the presence of adaptive immunity related genes (including RAG, histocompatibility genes and immune type receptors) previously thought to be restricted to the *Gnathostomata* (20–25, 63). The presence of lymphoid-like cells in lower chordates (17–19) further suggests that the lymphoid genetic toolbox was present before the emergence of humoral immunity. Accordingly, the E-protein dependence of the early lymphoid program suggest that the appearance of the full *Gnathostomata* E-protein repertoire was crucial for reinforcing the gene regulatory circuits that drove the emergence and expansion of the hematopoietic lineages constituting humoral immunity.

## Data Availability

Raw sequencing data are available from the European Nucleotide Archive (ENA) under accession number PRJEB29568 (E-protein knock-out RNAseq and E-protein ChIPseq) and PRJEB20316 (wild-type C57bl/6 RNAseq data).

## Author Contributions

TB, CG, and RM: planned the study; TB, SK, XL, AK, MH, CG, KS, and RM: performed the experiments; TB, LP-P, SK, XL, MH, AD, CG, KS, and RM: analyzed the data; CS and DH: provided critical insights; CS and RM: supervised the research; TB, LP-P, CG, and RM: wrote the manuscript. All authors reviewed the manuscript before submission.

### Conflict of Interest Statement

The authors declare that the research was conducted in the absence of any commercial or financial relationships that could be construed as a potential conflict of interest.

## References

[1] Flajnik MF Kasahara M. Origin and evolution of the adaptive immune system: genetic events and selective pressures. Nat Rev Genet. (2010) 11:47–59. 10.1038/nrg2703 19997068 PMC3805090

[2] Litman GW Rast JP Fugmann SD. The origins of vertebrate adaptive immunity. Nat Rev Immunol. (2010) 10:543–53. 10.1038/nri2807 20651744 PMC2919748

[3] Parra D Takizawa F Sunyer JO. Evolution of B cell immunity. Ann Rev Anim Biosci. (2013) 1:65–97. 10.1146/annurev-animal-031412-103651 25340015 PMC4203447

[4] Azevedo Portilho N Kobayashi M Yoshimoto M. What do the lineage tracing studies tell us? Consideration for hematopoietic stem cell origin, dynamics, and leukemia-initiating cells. Int J Hematol. (2018) 109:35–40. 10.1007/s12185-018-2537-9 30264284 PMC6450395

[5] Adolfsson J Månsson R Buza-Vidas N Hultquist A Liuba K Jensen CT. Identification of Flt3^+^ lympho-myeloid stem cells lacking erythro-megakaryocytic potential: a revised road map for adult blood lineage commitment. Cell. (2005) 121:295–306. 10.1016/j.cell.2005.02.013 15851035

[6] Månsson R Hultquist A Luc S Yang L Anderson K Kharazi S. Molecular evidence for hierarchical transcriptional lineage priming in fetal and adult stem cells and multipotent progenitors. Immunity. (2007) 26:407–19. 10.1016/j.immuni.2007.02.013 17433729

[7] Kondo M Weissman IL Akashi K. Identification of clonogenic common lymphoid progenitors in mouse bone marrow. Cell. (1997) 91:661–72. 10.1016/S0092-8674(00)80453-5 9393859

[8] Inlay MA Bhattacharya D Sahoo D Serwold T Seita J Karsunky H. Ly6d marks the earliest stage of B-cell specification and identifies the branchpoint between B-cell and T-cell development. Genes Dev. (2009) 23:2376–81. 10.1101/gad.1836009 19833765 PMC2764492

[9] Mansson R Zandi S Welinder E Tsapogas P Sakaguchi N Bryder D. Single-cell analysis of the common lymphoid progenitor compartment reveals functional and molecular heterogeneity. Blood. (2010) 115:2601–9. 10.1182/blood-2009-08-236398 19996414

[10] Mansson R Welinder E Åhsberg J Lin YC Benner C Glass CK. Positive intergenic feedback circuitry, involving EBF1 and FOXO1, orchestrates B-cell fate. Proc Natl Acad Sci USA. (2012) 109:21028–33. 10.1073/pnas.1211427109 23213261 PMC3529039

[11] Zlotoff DA Bhandoola A. Hematopoietic progenitor migration to the adult thymus. Ann N Y Acad Sci. (2011) 1217:122–38. 10.1111/j.1749-6632.2010.05881.x 21251013 PMC3076003

[12] Akashi K Traver D Miyamoto T Weissman IL. A clonogenic common myeloid progenitor that gives rise to all myeloid lineages. Nature. (2000) 404:193–7. 10.1038/35004599 10724173

[13] Pronk CJH Bryder D. Flow Cytometry-Based Identification of Immature Myeloerythroid Development. In: Hawley TS Hawley RG, editors. Flow Cytometry Protocols. Totowa, NJ: Humana Press (2011). p. 275–93. 10.1007/978-1-61737-950-5_13 21116988

[14] Manz MG Traver D Miyamoto T Weissman IL Akashi K. Dendritic cell potentials of early lymphoid and myeloid progenitors. Blood. (2001) 97:3333–41. 10.1182/blood.V97.11.3333 11369621

[15] Nappi AJ Ottaviani E. Cytotoxicity and cytotoxic molecules in invertebrates. BioEssays. (2000) 22:469–80. 10.1002/(SICI)1521-1878(200005)22:5<469::AID-BIES9>3.0.CO;2-4 10797487

[16] Svoboda O Bartunek P. Origins of the vertebrate erythro/megakaryocytic system. BioMed Res Int. (2015) 2015:10. 10.1155/2015/632171 26557683 PMC4628740

[17] Suzuki T Shin-I T Kohara Y Kasahara M. Transcriptome analysis of hagfish leukocytes: a framework for understanding the immune system of jawless fishes. Dev Comp Immunol. (2004) 28:993–1003. 10.1016/j.dci.2004.04.005 15236930

[18] Peddie CM Smith VJ ‘Lymphocyte-like’cells in ascidians: precursors for vertebrate lymphocytes? Fish Shellf Immunol. (1995) 5:613–29.

[19] Huang G Xie X Han Y Fan L Chen J Mou C The identification of lymphocyte-like cells and lymphoid-related genes in amphioxus indicates the twilight for the emergency of adaptive immune system. PLoS ONE. (2007) 2:e206 10.1371/journal.pone.0000206 17299586 PMC1784065

[20] Kapitonov VV Koonin EV. Evolution of the RAG1-RAG2 locus: both proteins came from the same transposon. Biol Direct. (2015) 10:20. 10.1186/s13062-015-0055-8 25928409 PMC4411706

[21] Morales Poole JR Huang SF Xu A Bayet J Pontarotti P. The RAG transposon is active through the deuterostome evolution and domesticated in jawed vertebrates. Immunogenetics. (2017) 69:391–400. 10.1007/s00251-017-0979-5 28451741

[22] Azumi K De Santis R De Tomaso A Rigoutsos I Yoshizaki F Pinto MR Genomic analysis of immunity in a Urochordate and the emergence of the vertebrate immune system: “waiting for Godot”. Immunogenetics. (2003) 55:570–81. 10.1007/s00251-003-0606-5 14530883

[23] Voskoboynik A Newman AM Corey DM Sahoo D Pushkarev D Neff NF. Identification of a colonial chordate histocompatibility gene. Science. (2013) 341:384–7. 10.1126/science.1238036 23888037 PMC3810301

[24] Du Pasquier L Zucchetti I De Santis R. Immunoglobulin superfamily receptors in protochordates: before RAG time. Immunol Rev. (2004) 198:233–48. 10.1111/j.0105-2896.2004.00122.x 15199966

[25] Prada JAH Haire RN Allaire M Jakoncic J Stojanoff V Cannon JP Ancient evolutionary origin of diversified variable regions demonstrated by crystal structures of an immune-type receptor in amphioxus. Nat Immunol. (2006) 7:875–82. 10.1038/ni1359 16799561 PMC3707131

[26] Arendt D Musser JM Baker CVH Bergman A Cepko C Erwin DH. The origin and evolution of cell types. Nat Rev Genet. (2016) 17:744–57. 10.1038/nrg.2016.127 27818507

[27] Simionato E Ledent V Richards G Thomas-Chollier M Kerner P Coornaert D. Origin and diversification of the basic helix-loop-helix gene family in metazoans: insights from comparative genomics. BMC Evol Biol. (2007) 7:33. 10.1186/1471-2148-7-33 17335570 PMC1828162

[28] Massari ME Murre C. Helix-Loop-Helix proteins: regulators of transcription in eucaryotic organisms. Mol Cell Biol. (2000) 20:429–40. 10.1128/MCB.20.2.429-440.2000 10611221 PMC85097

[29] Shain DH Neuman T Zuber MX. Embryonic expression and evolution of duplicated E-protein genes in *Xenopus laevis*: parallels with ancestral E-protein genes. Genetics. (1997) 146:345–53. 9136023 10.1093/genetics/146.1.345 PMC1207949

[30] Diez-Roux G Banfi S Sultan M Geffers L Anand S Rozado D. A high-resolution anatomical atlas of the transcriptome in the mouse embryo. PLoS Biol. (2011) 9:e1000582. 10.1371/journal.pbio.1000582 21267068 PMC3022534

[31] Semerad CL Mercer EM Inlay MA Weissman IL Murre C. E2A proteins maintain the hematopoietic stem cell pool and promote the maturation of myelolymphoid and myeloerythroid progenitors. Proc Natl Acad Sci USA. (2009) 106:1930–5. 10.1073/pnas.0808866106 19181846 PMC2644141

[32] Welinder E Mansson R Mercer EM Bryder D Sigvardsson M Murre C. The transcription factors E2A and HEB act in concert to induce the expression of FOXO1 in the common lymphoid progenitor. Proc Natl Acad Sci USA. (2011) 108:17402–7. 10.1073/pnas.1111766108 21972416 PMC3198373

[33] Yang Q Kardava L St.Leger A Martincic K Varnum-Finney B Bernstein ID. E47 controls the developmental integrity and cell cycle quiescence of multipotential hematopoietic progenitors. J Immunol. (2008) 181:5885–94. 10.4049/jimmunol.181.9.5885 18941177 PMC2597624

[34] Yang Q Esplin B Borghesi L E47 regulates hematopoietic stem cell proliferation and energetics but not myeloid lineage restriction. Blood. (2011) 117:3529–38. 10.1182/blood-2010-07-297689 21273306 PMC3072876

[35] Santos PM Ding Y Borghesi L. Cell-intrinsic *in vivo* requirement for the E47–p21 pathway in long-term hematopoietic stem cells. J Immunol. (2014) 192:160–8. 10.4049/jimmunol.1302502 24259504 PMC3893818

[36] Barndt RJ Dai M Zhuang Y. Functions of E2A-HEB heterodimers in T-cell development revealed by a dominant negative mutation of HEB. Molecul Cell Biol. (2000) 20:6677–85. 10.1128/MCB.20.18.6677-6685.2000 10958665 PMC86175

[37] Beck K Peak MM Ota T Nemazee D Murre C. Distinct roles for E12 and E47 in B cell specification and the sequential rearrangement of immunoglobulin light chain loci. J Exp Med. (2009) 206:2271–84. 10.1084/jem.20090756 19752184 PMC2757879

[38] Dias S Månsson R Gurbuxani S Sigvardsson M Kee BL. E2A proteins promote development of lymphoid-primed multipotent progenitors. Immunity. (2008) 29:217–27. 10.1016/j.immuni.2008.05.015 18674933 PMC2600583

[39] Ikawa T Kawamoto H Goldrath AW Murre C. E proteins and notch signaling cooperate to promote T cell lineage specification and commitment. J ExpMed. (2006) 203:1329–42. 10.1084/jem.20060268 16682500 PMC2121213

[40] Kwon K Hutter C Sun Q Bilic I Cobaleda C Malin S. Instructive role of the transcription factor E2A in early B lymphopoiesis and germinal center B cell development. Immunity. (2008) 28:751–62. 10.1016/j.immuni.2008.04.014 18538592

[41] Lin YC Jhunjhunwala S Benner C Heinz S Welinder E Mansson R. A global network of transcription factors, involving E2A, EBF1 and Foxo1, that orchestrates B cell fate. Nat Immunol. (2010) 11:635–43. 10.1038/ni.1891 20543837 PMC2896911

[42] Takeuchi A Yamasaki S Takase K Nakatsu F Arase H Onodera M E2A and HEB Activate the pre-TCRα promoter during immature T cell development. J Immunol. (2001) 167:2157–63. 10.4049/jimmunol.167.4.2157 11490000

[43] Wöhner M Tagoh H Bilic I Jaritz M Poliakova DK Fischer M. Molecular functions of the transcription factors E2A and E2-2 in controlling germinal center B cell and plasma cell development. J Exp Med. (2016) 213:1201–21. 10.1084/jem.20152002 27261530 PMC4925024

[44] Wojciechowski J Lai A Kondo M Zhuang Y. E2A and HEB are required to block thymocyte proliferation prior to pre-TCR expression. J Immunol. (2007) 178:5717–26. 10.4049/jimmunol.178.9.5717 17442955 PMC2265380

[45] Xu W Carr T Ramirez K McGregor S Sigvardsson M Kee BL. E2A transcription factors limit expression of Gata3 to facilitate T lymphocyte lineage commitment. Blood. (2013) 121:1534–42. 10.1182/blood-2012-08-449447 23297135 PMC3587319

[46] Zhuang Y Cheng P Weintraub H. B-lymphocyte development is regulated by the combined dosage of three basic helix-loop-helix genes, E2A, E2-2, and HEB. Molecul Cell Biol. (1996) 16:2898–905. 10.1128/MCB.16.6.2898 8649400 PMC231283

[47] Borghesi L Aites J Nelson S Lefterov P James P Gerstein R. E47 is required for V(D)J recombinase activity in common lymphoid progenitors. J Exp Med. (2005) 202:1669–77. 10.1084/jem.20051190 16365147 PMC2212960

[48] de Boer J Williams A Skavdis G Harker N Coles M Tolaini M. Transgenic mice with hematopoietic and lymphoid specific expression of Cre. Ear J Immunol. (2003) 33:314–25. 10.1002/immu.200310005 12548562

[49] Bergqvist I Eriksson M Saarikettu J Eriksson B Corneliussen B Grundström T. The basic helix-loop-helix transcription factor E2–2 is involved in T lymphocyte development. Eur J Immunol. (2000) 30:2857–63. 10.1002/1521-4141(200010)30:10<2857::AID-IMMU2857>3.0.CO;2-G 11069067

[50] Pan L Hanrahan J Li J Hale LP Zhuang Y. An analysis of T cell intrinsic roles of E2A by conditional gene disruption in the thymus. J Immunol. (2002) 168:3923–32. 10.4049/jimmunol.168.8.3923 11937548

[51] Zerbino DR Achuthan P Akanni W Amode MR Barrell D Bhai J. Ensembl 2018. Nucleic Acids Res. (2018) 46:D754–61. 10.1093/nar/gkx1098 29155950 PMC5753206

[52] Kumar S Stecher G Tamura K. MEGA7: molecular evolutionary genetics analysis version 7.0 for bigger datasets. Molecul Biol Evol. (2016) 33:1870–4. 10.1093/molbev/msw054 27004904 PMC8210823

[53] Dobin A Davis CA Schlesinger F Drenkow J Zaleski C Jha S. STAR: ultrafast universal RNA-seq aligner. Bioinformatics. (2013) 29:15–21. 10.1093/bioinformatics/bts635 23104886 PMC3530905

[54] Heinz S Benner C Spann N Bertolino E Lin YC Laslo P. Simple combinations of lineage-determining transcription factors prime cis-regulatory elements required for macrophage and B cell identities. Molecul Cell. (2010) 38:576–89. 10.1016/j.molcel.2010.05.004 20513432 PMC2898526

[55] Hayashi S McMahon AP. Efficient recombination in diverse tissues by a tamoxifen-inducible form of Cre: a tool for temporally regulated gene activation/inactivation in the mouse. Dev Biol. (2002) 244:305–18. 10.1006/dbio.2002.0597 11944939

[56] Gustafsson C De Paepe A Schmidl C Månsson R. High-throughput ChIPmentation: freely scalable, single day ChIPseq data generation from very low cell-numbers. BMC Genom. (2019) 20:59. 10.1186/s12864-018-5299-0 30658577 PMC6339279

[57] Langmead B Salzberg SL. Fast gapped-read alignment with Bowtie 2. Nature Methods. (2012) 9:357. 10.1038/nmeth.1923 22388286 PMC3322381

[58] Schrankel CS Solek CM Buckley KM Anderson MK Rast JP. A conserved alternative form of the purple sea urchin HEB/E2-2/E2A transcription factor mediates a switch in E-protein regulatory state in differentiating immune cells. Dev Biol. (2016) 416:149–61. 10.1016/j.ydbio.2016.05.034 27265865

[59] Cisse B Caton ML Lehner M Maeda T Scheu S Locksley R. Transcription factor E2-2 is an essential and specific regulator of plasmacytoid dendritic cell development. Cell. (2008) 135:37–48. 10.1016/j.cell.2008.09.016 18854153 PMC2631034

[60] Quong MW Martensson A Langerak AW Rivera RR Nemazee D Murre C. Receptor editing and marginal zone B cell development are regulated by the helix-loop-helix protein, E2A. J Exp Med. (2004) 199:1101–12. 10.1084/jem.20031180 15078898 PMC2211894

[61] Wikstrom I Forssell J Goncalves M Colucci F Holmberg D. E2-2 regulates the expansion of pro-B cells and follicular versus marginal zone decisions. J Immunol. (2006) 177:6723–9. 10.4049/jimmunol.177.10.6723 17082585

[62] Han Y Huang G Zhang Q Yuan S Liu J Zheng T. The primitive immune system of amphioxus provides insights into the ancestral structure of the vertebrate immune system. Dev Compar Immunol. (2010) 34:791–6. 10.1016/j.dci.2010.03.009 20363248

[63] Kasahara M Suzuki T Pasquier LD. On the origins of the adaptive immune system: novel insights from invertebrates and cold-blooded vertebrates. Trends Immunol. (2004) 25:105–11. 10.1016/j.it.2003.11.005 15102370

[64] Meister M. Blood cells of drosophila: cell lineages and role in host defence. Curr Opin Immunol. (2004) 16:10–5. 10.1016/j.coi.2003.11.002 14734104

[65] Pancer Z. Dynamic expression of multiple scavenger receptor cysteine-rich genes in coelomocytes of the purple sea urchin. Proc Natl Acad Sci USA. (2000) 97:13156–61. 10.1073/pnas.230096397 11069281 PMC27194

[66] Zhang B Lin Y-Y Dai M Zhuang Y. Id3 and Id2 act as a dual safety mechanism in regulating the development and population size of innate-like γ*δ* T cells. J Immunol. (2014) 192:1055–63. 10.4049/jimmunol.1302694 24379125 PMC3899720

[67] Schmidl C Rendeiro AF Sheffield NC Bock C. ChIPmentation: fast, robust, low-input ChIP-seq for histones and transcription factors. Nat Methods. (2015) 12:963–5. 10.1038/nmeth.3542 26280331 PMC4589892

[68] Schmitz R Young RM Ceribelli M Jhavar S Xiao W Zhang M. Burkitt lymphoma pathogenesis and therapeutic targets from structural and functional genomics. Nature. (2012) 490:116–20. 10.1038/nature11378 22885699 PMC3609867

[69] Hikima J-i Cioffi CC Middleton DL Wilson MR Miller NW Clem LW. Evolution of Transcriptional Control of the IgH Locus: characterization, expression, and function of TF12/HEB homologs of the catfish. J Immunol. (2004) 173:5476–84. 10.4049/jimmunol.173.9.5476 15494495

[70] Ikawa T Kawamoto H Wright LYT Murre C. Long-term cultured E2A-deficient hematopoietic progenitor cells are pluripotent. Immunity. (2004) 20:349–60. 10.1016/S1074-7613(04)00049-4 15030778

[71] King AM Keating P Prabhu A Blomberg BB Riley RL. NK cells in the CD19– B220+ bone marrow fraction are increased in senescence and reduce E2A and surrogate light chain proteins in B cell precursors. Mech Ageing Dev. (2009) 130:384–92. 10.1016/j.mad.2009.03.002 19428458 PMC2743292

[72] Ghosh HS Cisse B Bunin A Lewis KL Reizis B. Continuous expression of the transcription factor E2-2 maintains the cell fate of mature plasmacytoid dendritic cells. Immunity. (2010) 33:905–16. 10.1016/j.immuni.2010.11.023 21145760 PMC3010277

[73] Grajkowska LT Ceribelli M Lau CM Warren ME Tiniakou I Nakandakari Higa S. Isoform-specific expression and feedback regulation of E protein TCF4 control dendritic cell lineage specification. Immunity. (2017) 46:65–77. 10.1016/j.immuni.2016.11.006 27986456 PMC5243153

[74] Nagasawa M Schmidlin H Hazekamp MG Schotte R Blom B. Development of human plasmacytoid dendritic cells depends on the combined action of the basic helix-loop-helix factor E2-2 and the Ets factor Spi-B. Eur J Immunol. (2008) 38:2389–400. 10.1002/eji.200838470 18792017

