# Supplementary material for: The Concerted Action of E2-2 and HEB Is Critical for Early Lymphoid Specification
Source: Front Immunol. 2019 Mar 18;10:455. doi: 10.3389/fimmu.2019.00455 (PMC6433000; doi:10.3389/fimmu.2019.00455)
Supplement: Supplementary file 3 [file Table_3.docx]

**Supplemental Materials and Methods**

**ChIPseq**

Antibodies

Per ChIP, 1.5μg of anti-E2A (Santa Cruz, cat# sc-349 X, Lot: G2911), 3μg of monoclonal anti-HEB (Santa Cruz Biotechnology, cat# sc-365980 X, lot# G0717) or 3ug of anti-E2-2 (FinTest, cat# FNab08553, lot#2017 12 05) was added to 30μl (E2A) or 10μl (HEB and E2-2) Protein G-coupled Dynabeads (ThermoFisher) in PBS with 0.5% BSA and incubated with rotation for 4h at 4°C. Antibody-coated Dynabeads were washed with PBS with 0.5% FCS before further use.

In addition, we also tested the following antibodies (all from Santa Cruz Biotechnology) that failed to give enrichment using our ChIP/ChIPmentation protocol and sequencing as readout:

E2A Antibody (Yae), cat# sc-416, lot F2717

E2A Antibody (G-9), cat# sc-365261 X, lot G0512

E2A Antibody (G-2), cat# sc-133075, lot G1417

HEB Antibody (D-3), cat# sc-28364, lot D2016

HEB Antibody (C-7), cat# sc-365572 X, lot C2811

EBF Antibody (C-8), cat# sc-137065, lot D1116

E2-2 ITF-2 Antibody (C-8), X cat# sc-393407 X, lot G1917

E2-2 ITF-2 Antibody (C-1), cat# sc-393255 X, lot A1414

E2-2 ITF-2 Antibody (G-8), X cat# sc-515325 X, lot A1018

E2-2 ITF-2 Antibody (367.2), cat# sc-101095, lot B1909

ITF-2 Antibody (C-8), cat# sc-393407, lot G1917

Although HEB sc-365980 X was chosen because of better performance, the following HEB antibody also gave satisfactory enrichment:

HEB Anti-TCF12 antibody, Abcam cat# ab70746, lot GR208569-7

E2A ChIPseq

5 million fixed cells were washed in Lysis buffer 1 (50mM Hepes/KOH, 140mM NaCl, 1mM EDTA, 10% glycerol, 0.5% NP-40, 0.25% Triton X-100 and 1x protein inhibitors), Lysis buffer 2 (10mM Tris/HCl, 200mM NaCl, 1mM EDTA, 0.5mM EGTA and 1x protein inhibitors) and resuspended in 500μl Lysis buffer 3 (10mM Tris/HCl, 100mM NaCl, 1mM EDTA, 0.5mM EGTA, 0.1% Na-Deoxycholate, 0.5% N-lauroylsarcosine and 1x protein inhibitors). Chromatin was fragmented by sonication with a microtip on ice/ice water (10 cycles, 10 seconds with 25% output). Membrane proteins were solubilized by adding 1% Triton-X and cell debris were removed by centrifugation. Cell lysates were then incubated overnight with antibody-coated Dynabeads. Samples were washed with low-salt buffer (50mM Tris/HCl, 150 mM NaCl, 0.1% SDS, 0.1% NaDOC, 1% Triton X-100, and 1mM EDTA), high-salt buffer (50mM Tris/HCl, 500mM NaCl, 0.1% SDS, 0.1% NaDoc, 1% Triton X-100, and 1 mM EDTA) and LiCl buffer (10 mM Tris/HCl, 250 mM LiCl, 0.5% IGEPAL CA-630, 0.5% NaDOC, and 1mM EDTA), followed by two washes with TE buffer (10mM Tris/HCl and 1mM EDTA). Samples were diluted with 200μl ChIP elution buffer (10mM Tris/HCl, 0.5% SDS, 300mM NaCl, and 5 mM EDTA) and 2μl of 20μg/ml proteinase K (Thermo Scientific), followed by incubation shaking overnight at 65°C. After reverse crosslinking, 1μl 4mg/ml RNase was added and incubated at 37°C for 30min. After another 2h of incubation with 2μl of proteinase K (10mg/ml) at 55°C, samples were placed in a magnet to trap magnetic beads and supernatants were collected. DNA purification was carried out using Qiagen QIAquick PCR Purification Kit. 2ng of DNA per sample was used for library preparation using the ThruPLEX DNA-seq kit (Rubicon Genomics) with 11 cycles of PCR amplification.

Material for input controls (non-immunoprecipitated DNA) was set aside after sonication and subsequently reverse cross-linked and library prepped in an identical manner to the E2A ChIPseq libraries.

HEB and E2-2 high-througput ChIPmentation

2 million fixed cells were thawed at room temperature, pelleted and diluted with SDS lysis buffer (50mM Tris/HCl pH8, 0.5% SDS and 10mM EDTA pH8) and placed at 4°C for 15 minutes. Cells were sonicated for 12 cycles of 30 sec on/30 sec off at high power using a Bioruptor Plus (Diagenode). To neutralize the SDS, Triton X100 was added to a final concentration of 1% along with 2μl 50x cOmplete protease inhibitor (final 1x). Samples were incubated at room temperature for 10min. Antibody-coated Dynabeads were washed with PBS with 0.5% FCS and mixed with cell lysate in PCR tubes. Tubes were incubated rotating overnight at 4°C. Immuno-precipitated chromatin was washed with low-salt, high-salt and LiCl buffer as for the E2A ChIP. The two washes with TE buffer were followed by two washes with ice-cold Tris/HCl pH8. For tagmentation, bead bound chromatin was resuspended in 30μl of tagmentation buffer, 1μl of transposase (Nextera, Illumina) was added and samples were incubated at 37°C for 10 minutes followed by two washes with low-salt buffer. Bead bound tagmented chromatin was diluted in 30μl of water. 15 μl PCR master mix (Nextera, Illumina) and 5 μl indexed amplification primers[^1^](#_ENREF_1) (0.125uM final concentration) were added and libraries amplified without prior DNA purification using the following program (as per the high-throughput ChIPmentation approach [^2^](#_ENREF_2)): 72^°^C 5min (adapter extension); 95^°^C 5min (reverse cross-linking); followed by 11 cycles of 98^°^C 10s, 63^°^C 30s and 72^°^C 3min (amplification).

Library cleanup and sequencing

After PCR amplification, cleanup of all libraries was done using Agencourt AmPureXP beads (Beckman Coulter) at a ratio of 1:1. DNA concentrations in purified samples were measured using the Qubit dsDNA HS Kit (Invitrogen). Libraries were single-end sequenced using Illumina’s HiSeq2000 sequencing system (E2A, read length 37 cycles) or Nextseq500 platform (HEB and E2-2, read length 50 cycles).

**Bioinformatics**

QC and basic read processing

Sample quality was evaluated with FastQC (v0.11.15). Trim Galore (v.0.4.0) and Cutadapt (v.1.9.1) were used with default parameters in order to trim reads before mapping.

RNAseq analysis

STAR (v2.5.2b) (<https://github.com/alexdobin/STAR>) was used with default settings for paired-end-reads to map the samples to mm10 genome. Filtered tag directories were made with HOMER (v4.6) (http://homer.ucsd.edu/homer/) using the *makeTagDirectory* command with the strand specific pair-end read parameter (*-sspe*) and restricting the tags per base to 3 (*-tbp3*) to remove reads resulting from PCR over-amplification.

HOMER’s *analyzeRepeats.pl* was used to count the exonic reads in the sense direction of the gene. To assess differential gene expression EdgeR was used on raw read counts. To consider a gene as differentially expressed an adjusted p-value < 0.01 and a twofold change in expression was required. Genes with > 30 reads in at least 2 of the analysed samples were considered expressed and included in the analysis.

RNAseq read counts were Log10 quantile normalized with R (v3.3.3) for PCA analysis and display in heatmaps. Metascape (<http://metascape.org>), was used to produce heatmaps with the enriched gene ontology terms of differentially expressed genes.

ChIPseq analysis

The reads were mapped to the mm10 genome using bowtie2 (v. 2.3.3.1) with default parameters. Filtered tag directories were made with HOMER (v4.6) (http://homer.ucsd.edu/homer/) using the *makeTagDirectory* command with restricting the tags per base to 1 (*-tbp1*) to remove reads resulting from PCR over-amplification.

For visualization using the UCSC genome browser (<https://genome.ucsc.edu>), total reads were normalized to 10 million and tracks prepared using HOMER’s *makeUCSCfile*.

Peak finding was performed from filtered tag directories using HOMER’s *findPeaks* with settings to obtain narrow peaks (*-size 100*) with variable peak width (*-region*) not present in the input control (*-i -inputSize4 -F 8)*.

For identifying peak overlaps, identified peaks were merged using HOMER’s *mergePeaks* based on actual positions (*-d given*).

To analyse subsets of peaks containing instances of HEB and E2A DNA binding motifs these were identified in the genome and overlapped with peaks using HOMER’s *annotatePeaks.pl* specifying the position weight matrixes of interest (*-m*).

Motif enrichment analysis was performed by means of HOMER’s *findMotifsGenome.pl*, considering the variable size (*-size given*) of the peaks identified.

**Supplementary references**

1 Buenrostro, J. D., Wu, B., Chang, H. Y. & Greenleaf, W. J. ATAC-seq: A Method for Assaying Chromatin Accessibility Genome-Wide. *Current protocols in molecular biology* **109**, 21.29.21-21.29.29, doi:10.1002/0471142727.mb2129s109 (2015).

2 Gustafsson, C., De Paepe, A., Schmidl, C. & Månsson, R. High-throughput ChIPmentation: freely scalable, single day ChIPseq data generation from very low cell-numbers. *BMC Genomics* **20**, 59, doi:10.1186/s12864-018-5299-0 (2019).
