# Supplementary material for: The Concerted Action of E2-2 and HEB Is Critical for Early Lymphoid Specification
Source: Front Immunol. 2019 Mar 18;10:455. doi: 10.3389/fimmu.2019.00455 (PMC6433000; doi:10.3389/fimmu.2019.00455)
Supplement: Supplementary file 6 [file Image_1.pdf]

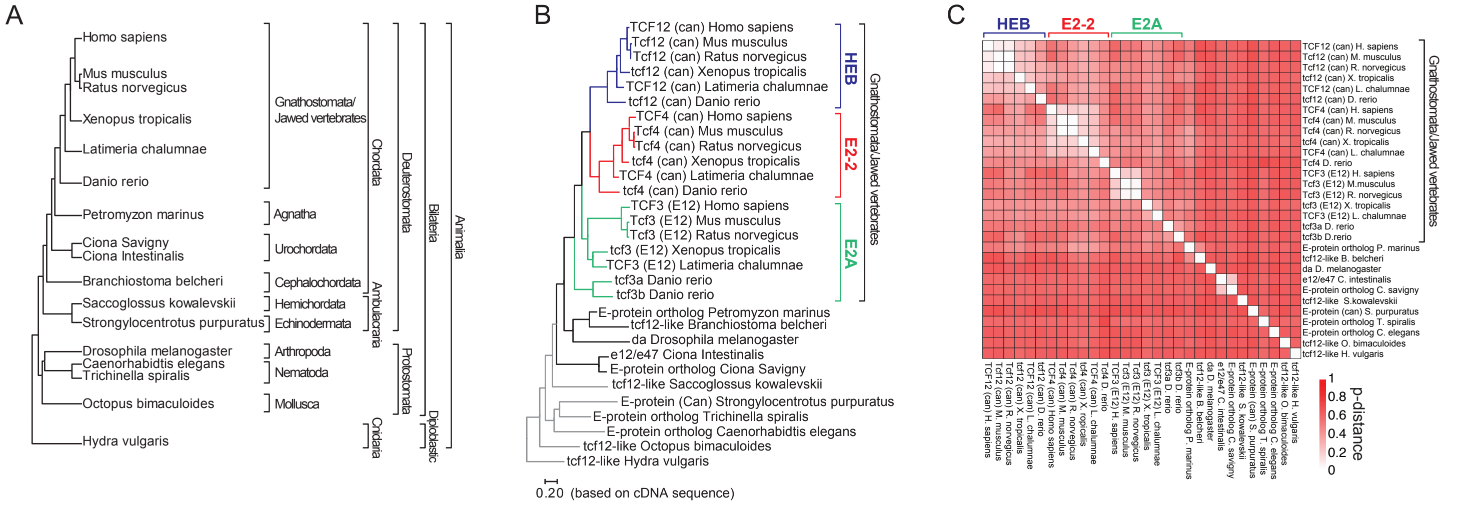

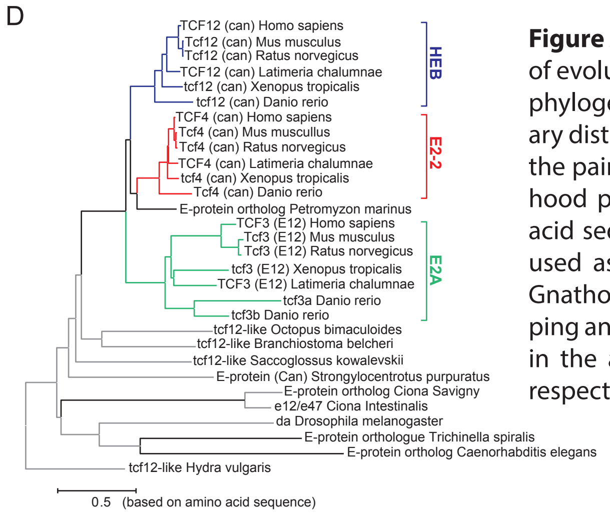

Figure S1. Phylogenetic analysis of the E-protein family. (A) Schematic overview of evolutionary relationships and classification of animals included in the E-protein phylogenetic analysis. (B) Maximum Likelihood phylogenetic tree of the evolutionary distances between E-protein cDNA sequences in Bilateria. (C) Matrix displaying the pairwise p-distances between analyzed cDNA sequences. (D) Maximum Likelihood phylogenetic tree of the evolutionary distances between E-protein amino acid sequences in Bilateria. For the analysis in panel B and D: Hydra vulgaris was used as an out-group for the analysis; colors indicate the E-protein families in Gnathostomata; and grey indicates branches with low support in the bootstrapping analysis (<70% of trees generated maintain the branches). For sequences used in the analysis and alignments, see Table S1 and Supplemental data sheet 1-2 respectively.

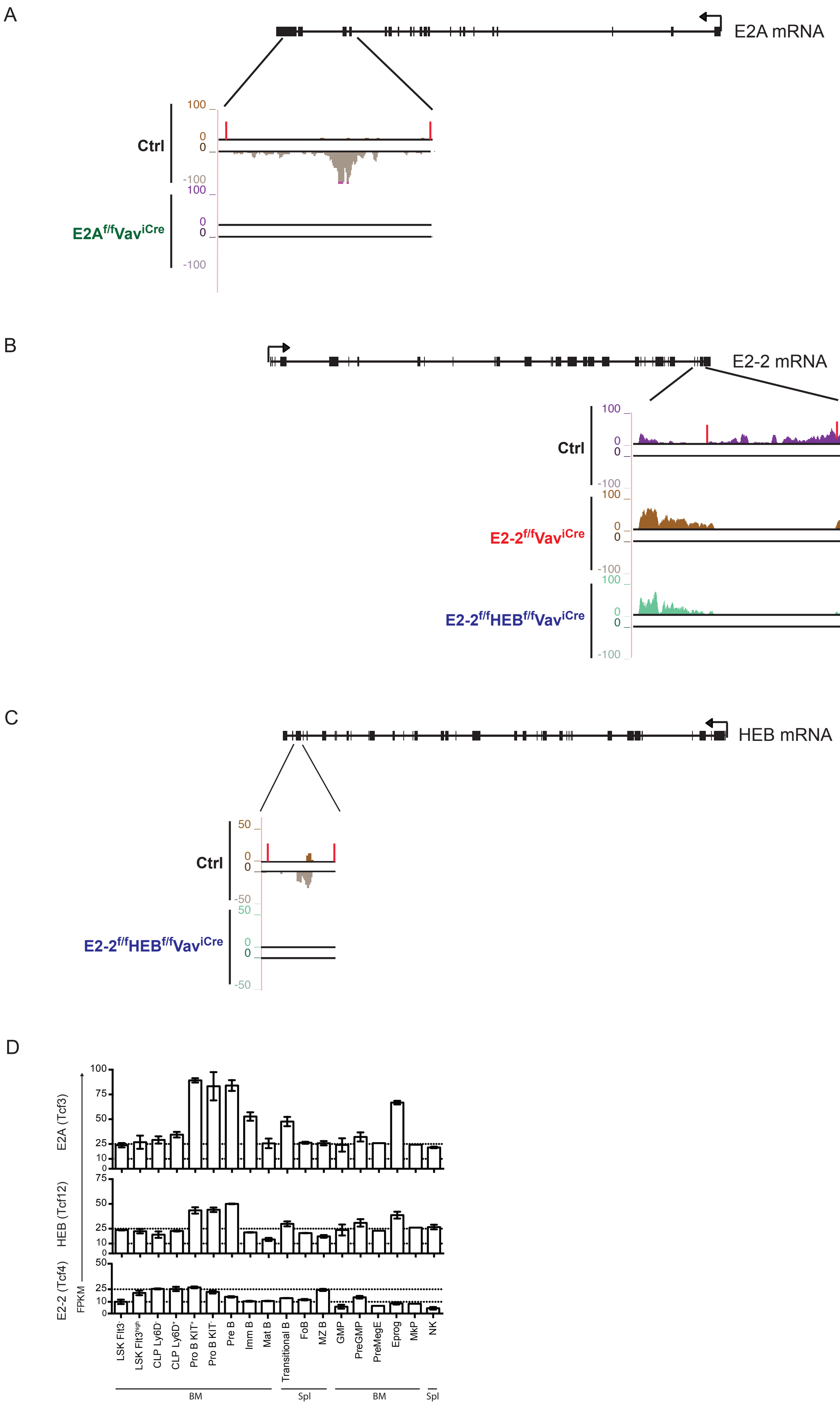

Figure S2. Expression pattern and deletion of floxed exons of E2A, E2-2 and HEB. (A-C) RNA expression of the floxed E-protein exons in mice with indicated genotypes. Locations of loxP sites are indicated with red lines. (D) Expression (RPKM) of the E-proteins in indicated cell types from bone marrow (BM) and spleen (Spl).

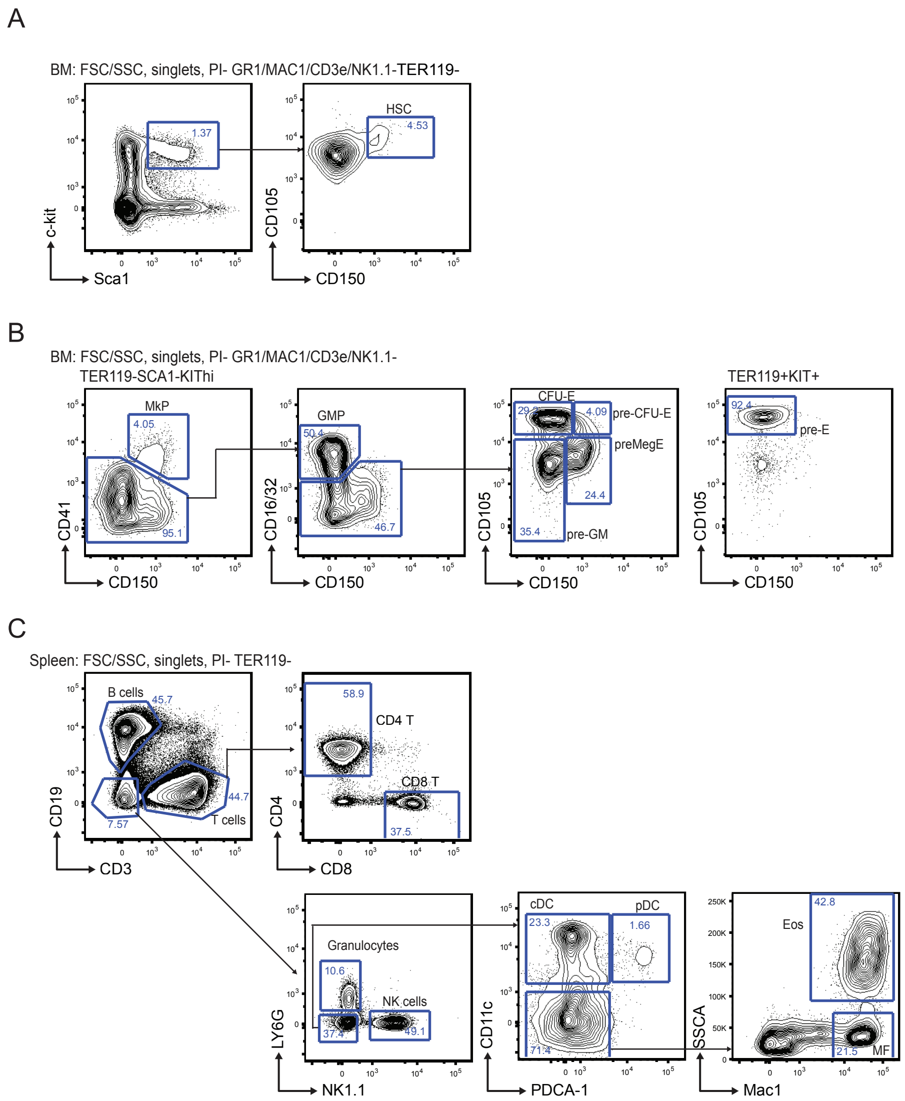

Figure S3. Gating strategies for identification of hematopoietic cells. Gating strategy for the identification of (A) hematopoietic stem cells (HSCs) and (B) erythro-myeloid progenitors (B) in bone marrow. (C) Gating strategy for the identification of mature hematopoietic cell types (CD4/8 T-cells, CD4/8 T; granulocytes, Gr; NK-cells, NK; conventional dendritic cells, cDC; plasmacytoid dendritic cells, pDC; eosinophiles, Eos; and macrophages, MF) in spleen.

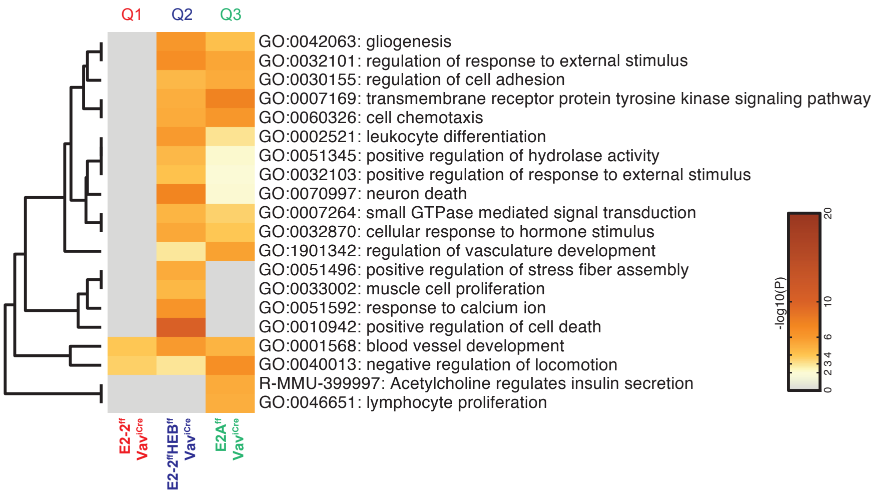

Figure S4. Functional annotation of genes differentially expressed in E-protein deficient animals. Metascape (gene ontology) analysis of genes with significantly differential expression (adjusted p-value <0.01, Q1-3 in Fig. 5B) in LY6D- CLPs from the indicated genotypes (as compared to LY6D- CLPs from control animals).

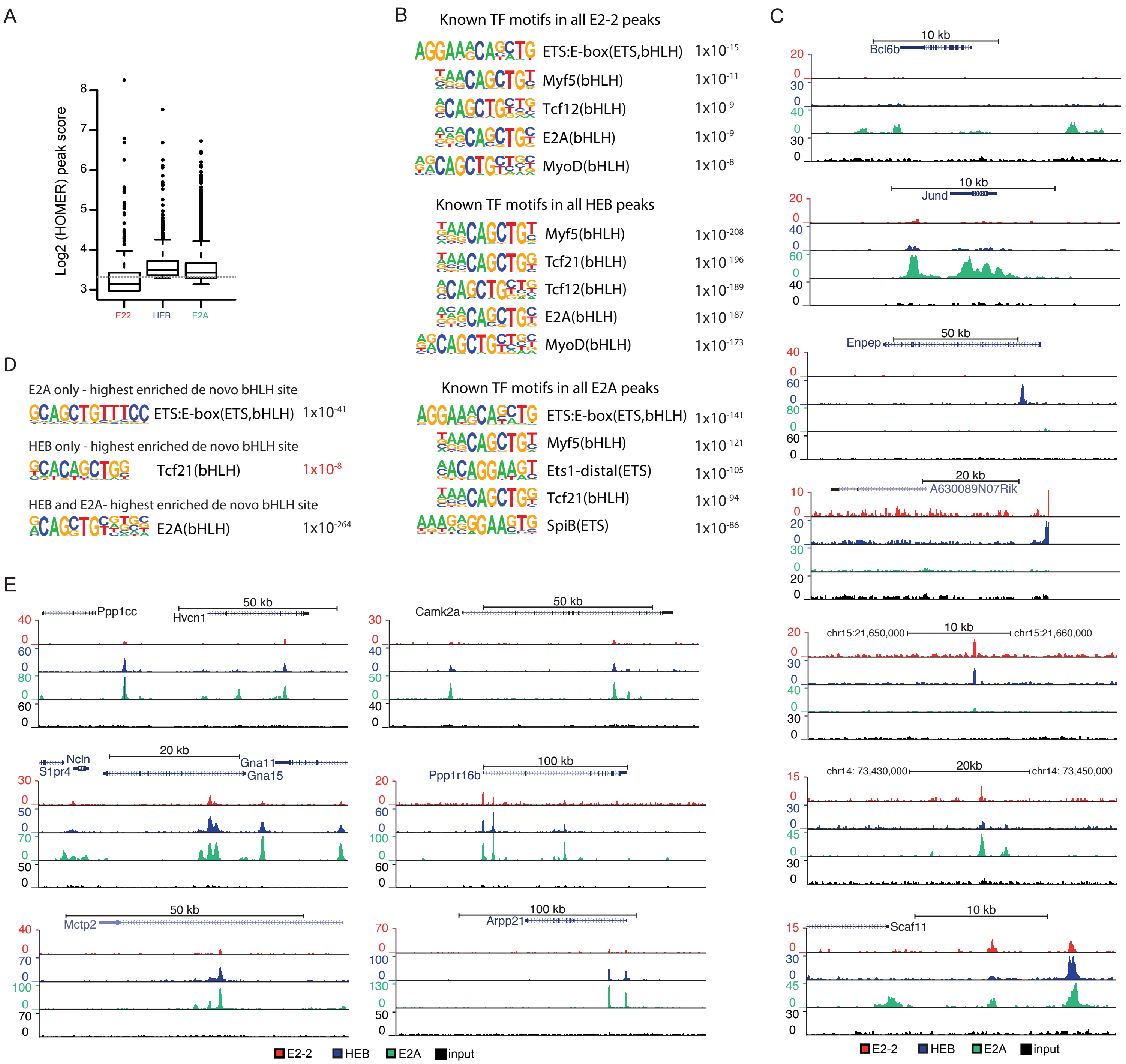

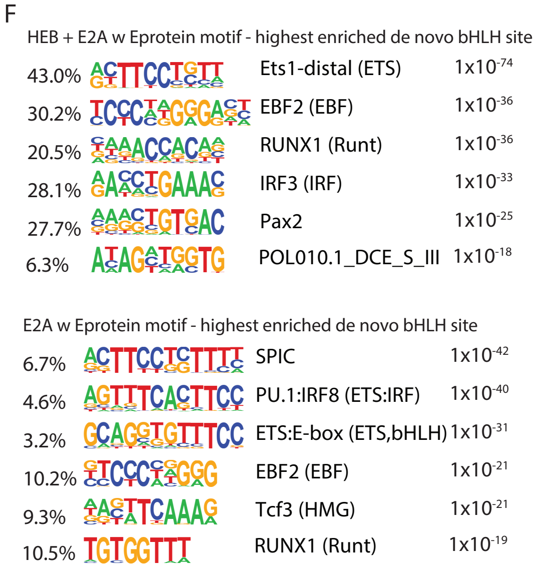

Figure S5. Enrichment of transcription factor binding motifs in E2A and HEB bound regions. (A) Peak scores (HOMER peak quality score) of all identified E2-2, HEB and E2A peaks before filtering on peak score. Only peaks with peak score ≥10 were considered for further analysis. The peak score cut-off is indicated with a grey dotted line. (B) Enrichment of known transcription factor binding sites in E2-2, HEB and E2A peaks. Top five most significantly enriched binding motifs are shown together with their corresponding enrichment p-value. (C) Examples of regions displaying biased E-protein binding. (D) De novo motif enrichment analysis of regions displaying combined E-protein (HEB and E2A) binding, only E2A binding or only HEB binding. The top significantly enriched bHLH motif identified is displayed together with the enrichment p-value. E2-2 binding was not considered, as the number of regions is too low to perform relevant motif enrichment analysis. (E) Genome browser tracks showing E-protein near central B-lineage genes. (F) De novo motif enrichment analysis of regions containing bHLH motifs that display combined E-protein binding or only E2A binding. Significantly enriched motifs are shown together with the corresponding enrichment p-value and the percentage of target sequences containing the identified motif.
